# The interplay between dendritic cells and CD8 T lymphocytes is a crucial component of SARS-CoV-2 immunity

**DOI:** 10.1038/s41423-020-00624-1

**Published:** 2021-01-08

**Authors:** Jonas Buttenschön, Jochen Mattner

**Affiliations:** 1 Mikrobiologisches Institut - Klinische Mikrobiologie, Immunologie und Hygiene, Universitätsklinikum Erlangen and Friedrich-Alexander Universität (FAU) Erlangen-Nürnberg, Erlangen, Germany; 2 Medical Immunology Campus Erlangen, FAU Erlangen-Nürnberg, Erlangen, Germany

**Keywords:** Biomarkers, Immunology

SARS-CoV-2, a novel beta-coronavirus (CoV), causes the coronavirus disease 2019 (COVID-19) pandemic, which manifests with a wide clinical spectrum ranging from asymptomatic carriage or mild disease to hospitalization and even death. The highly divergent and variable clinical outcomes, as well as the fluctuating involvement of different organ systems, link SARS-CoV-2 pathogenesis to the immune response of an individual host. Thus, detailed knowledge of the cellular and humoral mechanisms underlying antiviral immunity against SARS-CoV-2 is pivotal for understanding the concept of host susceptibility, evaluating therapeutic regimens and designing vaccination strategies. However, most studies so far have focused only on the analysis of immune responses in convalescent individuals. In contrast, Zhou and colleagues explored in detail different innate and adaptive immune cells in the peripheral blood of patients with acute disease in their latest report published in *Immunity*. Compared to convalescent individuals, they observed that acute SARS-CoV-2 infection quantitatively and qualitatively impairs dendritic cell (DC) and CD8 T-cell responses and interactions, despite rapid and abundant antibody production targeting dominant viral antigen epitopes.^1^

**Fig. 1:**
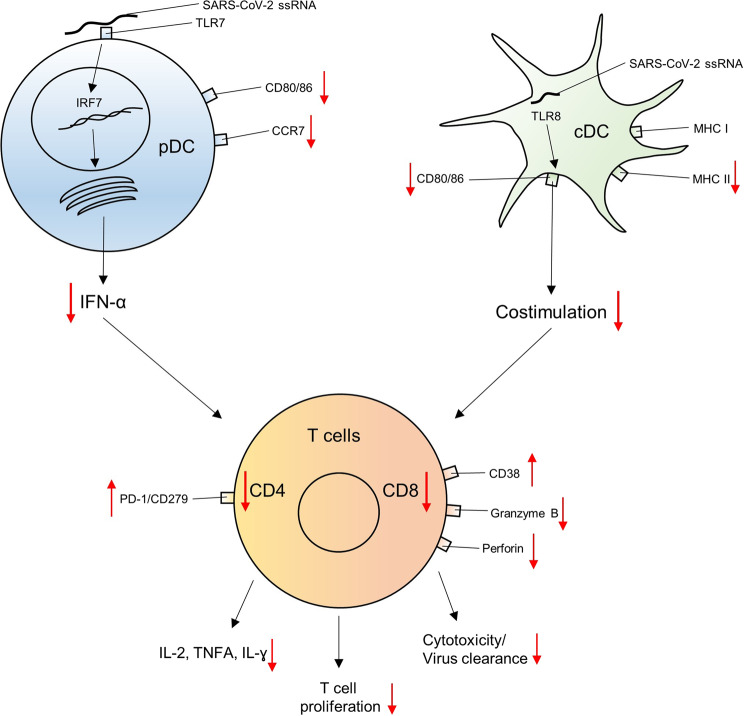
A numeric reduction and impaired interaction of DCs and T lymphocytes characterizes an acute infection with SARS-CoV-2. DCs release less type I interferon and express fewer chemokines and costimulatory molecules. Subsequently, the generation and expansion of SARS-CoV-2-specific CD4 and CD8 T cells is delayed, and the release of Th1 cytokines is impaired, resulting in enhanced viral replication

Host immune responses play a prominent role in immune pathogenesis and antiviral defense. SARS coronaviruses infect DCs^2^, among other cells, which significantly contribute to both innate and adaptive immunity.^3^ DCs, for example, efficiently prime CD8 T-cell responses through the cross-presentation of viral antigens. CD8 T cells themselves play a vital role in virus clearance and immunological memory. Indeed, among reported disruptions of different immune cell populations, low CD8 T-cell counts in particular are associated with an (hyper)inflammatory status in patients with COVID-19, disruption of tissue integrity and poor clinical outcome.^4^ Thus, intensive investigation is being directed at identifying the individual cellular and molecular mechanisms conferring protection against SARS-CoV-2 (Fig. 1).

Zhou and colleagues studied eight patients with severe and 33 with mild COVID-19 disease.^1^ All patients had severe disease required oxygen supplementation. Six individuals had acute severe disease and 11 acute mild disease; the remaining 24 individuals were included in the convalescent patient cohort. All patients received antiviral agents following hospital admission.^5^

The authors report a broad numerical reduction in peripheral immune cells including T lymphocytes, natural killer (NK) cells, DCs and monocytes in patients with acute disease compared to healthy donors. In addition, the relative distribution of conventional DCs (cDCs) compared to plasmacytoid DCs (pDCs) was significantly reduced in acute disease but particularly enhanced in convalescent patients. Conversely, significantly smaller numbers of myeloid-derived suppressor cells (M-MDSCs) were recovered from healthy donors. Importantly, M-MDSCs were the only cell population that significantly differed between patients with acute mild and acute severe disease. An accumulation of immature neutrophils and monocytes with an immunosuppressive profile in the blood and lungs of COVID-19 patients has also been recently reported.^6^ In addition, cDC to pDC ratios were increased in patients with acute severe compared to acute mild disease. DC and monocyte counts were even lower in convalescent patients than in healthy donors despite the recovery of NK and T cells. Unfortunately, no data on M-MDSCs in convalescent patients were provided.

Furthermore, in addition to numeric reductions, the functions of DCs were impaired, as evidenced by a reduced expression of the costimulatory molecules CD80 and CD86 in response to a “maturation cocktail” consisting of IL-6, TNFA and prostaglandin E2 (PGE2). Upon stimulation of Toll-like receptors (TLRs), the DCs from patients with acute disease did not properly upregulate HLA-DR, an MHC class II cell surface receptor, or CCR7, a chemokine that directs DCs to secondary lymphoid organs. Subsequently, these DCs triggered a significantly reduced proliferation of T cells in coculture. Moreover, type I interferons that are pivotal for antiviral responses^7^ were less significant or even not induced at all in acute disease and after convalescence.

The observed T-cell lymphopenia was associated with significantly reduced T-cell proliferation and cytokine production. CD4 and CD8 T lymphocytes originating from COVID-19 patients released significantly less IL-2, TNFA and IFN-γ upon T-cell engagement or polyclonal stimulation. CD4 T cells in both COVID-19 cohorts more frequently expressed PD-1, a marker of T-cell exhaustion. Conversely, increased expression of CD38, the cyclic ADP ribose hydrolase that metabolizes nicotinamide dinucleotide (NAD+) and regulates cell adhesion, signal transduction and calcium signaling,^8^ is suggested to be associated with enhanced activation of CD8 T cells in COVID-19 patients. However, neither marker, CD38 or PD-1, correlated with the disease severity. Other markers were not studied.

To assess viral antigen-specific humoral and cellular immunity, the authors investigated antibody and T-cell responses by ELISA, pseudotyped virus neutralization assays and ELISPOT. All eight patients with acute disease developed antibodies against the receptor-binding domain (RBD) and the nucleocapsid (NP) of SARS-CoV-2 within two weeks, independent of whether they had mild or severe disease. While all of the patients with mild disease developed NP-specific T-cell responses, only 50% of their T cells were reactive to RBD. In contrast, none of the patients with severe disease exhibited T cell-mediated immunity against RBD or NP. The patients with severe disease also exhibited higher viral loads, indicating that impaired T cell-mediated immunity perpetuates COVID-19 pathogenesis and viral replication.

Finally, the antibody and T-cell responses in 23 of the 24 covalescent patients were explored at ~30 days after the onset of symptoms. In all cases, IgG antibodies against RBD and NP developed. However, it remained unclear whether the antibodies detected in this cohort, similar to those in patients with acute disease, included neutralizing isotypes. In contrast, only 61% or 83% of cases developed RBD- or NP-specific T-cell responses, respectively. Interestingly, CD4 T cells responded more frequently and more robustly to these two antigens than CD8 T lymphocytes.

Considering these data and the fact that convalescent patients can lose antibodies after infection,^9^ it needs to be questioned whether antibodies are a suitable readout for determining immunogenicity in a vaccinated individual or in convalescent patients. Thus, SARS-CoV-2-specific T-cell responses might be the preferred readout for determining immunogenicity. Indeed, a recent study showed that SARS-CoV-2-specific memory CD8 T cells are detectable in convalescent individuals who are seronegative for anti-antibodies targeting the spike (S) and nucleoprotein (N) of SARS-CoV-2.^10^ However, it remains unclear whether convalescent patients experiencing reinfection with SARS-CoV-2 have lost cellular and/or humoral immunogenicity at the time of reinfection.

In conclusion, Zhou et al. report that acute SARS-CoV-2 infection results in a broad numeric reduction in many different immune cells in the peripheral blood. In particular, DC and CD8 T-cell functions were impaired (Fig. 1), while antibody responses were induced independent of the severity of disease.^1^ Invasive pulmonary aspergillosis,^11^ a fungal disease until now seen primarily in immunocompromised individuals, might reflect a consequence of the broad immune cell reduction in even immunocompetent patients with acute COVID-19 disease.^12^ Although these data suggest that DCs and CD8 T cells are pivotal for establishing immunity against SARS-CoV-2, further studies need to delineate in detail the impact of the observed DC and T-cell dysfunction on viral pathogenesis, clinical outcome and viral transmission. Furthermore, the timeframe of this immune cell depletion and its potential association with an increased risk for reinfection needs to be specified, as does the effect of comorbidities on immune cell function. In addition, the cellular source of inflammatory cytokines in the frequently observed hyperinflammatory syndrome in COVID-19 patients needs to be identified. Conversely, immunological readouts for evaluating the efficacy of vaccine candidates need to be carefully examined and should include the analysis of humoral immunity and the evaluation of cellular immune responses.
